# Time for tolerance: exploring the influence of learning institutions on the recognition of political rights among immigrants

**DOI:** 10.1186/s40878-018-0100-8

**Published:** 2018-12-05

**Authors:** Per Adman, Per Strömblad

**Affiliations:** 1 Department of Government, Uppsala University, Uppsala, Sweden; 2 Department of Political Science, Linnaeus University, Kalmar, Sweden

**Keywords:** Political tolerance, Learning institutions, Immigrants, Sweden

## Abstract

This paper empirically evaluates the idea that individual level political tolerance is influenced by the overall tolerance in a given society. The expectation is that more tolerant attitudes would be developed as a consequence of exposure to a social environment in which people in general are more inclined to accept freedom of speech, also when a specific message challenges one’s own values and beliefs. A theoretical learning model is formulated, according to which more broad-minded and permissive attitudes, from a democratic point of view, are adopted as a result of (1) an adjustment stimulated by mere observation of an overall high-level of political tolerance in society (‘passive learning’), and (2) an adjustment due to cognition and interaction within important spheres in society (‘active learning’). Using survey data, we explore empirically how length of residence among immigrants in the high-tolerance country of Sweden is related to measures of political tolerance. Further, we examine to what extent a time-related effect is mediated through participation in a set of ‘learning institutions’—focusing on activities related to education, working-life, civil society and political involvement. In concert with expectations, the empirical findings suggest that a positive effect of time in Sweden on political tolerance may be explained by a gradual adoption of the principle that political rights should be recognized. Importantly, however, such an adoption seems to require participation in activities of learning institutions, as we find that passive learning in itself is not sufficient.

## Introduction

Does an overall tolerant social environment serve as a seedbed, in which initially narrow-minded sentiments gradually wither away? In this paper, we explore differences in political tolerance, thus challenging assumptions of rigidity in the level of tolerance acquired early in life (e.g. Gibson, 2011, pp. 419–420). Utilizing a dynamic feature of cross-section data, our study aims to explore how possible mechanisms of ‘learning’ may explain differences between population categories, when it comes to the willingness to allow freedom of expression in contemporary multicultural democracies.

Political tolerance is habitually understood as the propensity of a person to support political rights for groups whose members share values and/or a way of life disliked by the same person (Marquart-Pyatt & Paxton, 2007; Stouffer, 1955; Sullivan, Piereson, & Marcus, 1982). In the abstract, it may be fairly comfortable to embrace principles of democratic privileges, such as freedom of speech for all citizens (or, yet more inclusive, for all members of given society). However, defending the right to, in actual practice, publicly express viewpoints that appear to be light-years away from one’s own may be considerably more demanding. It is much to expect from the devoted pro-choice activist, that s/he primarily will count the blessings of free speech when passing by an anti-abortion demonstration. Likewise, a Christian, who strongly believes that all people should conform to the norms of Bible verses condemning homosexuality, would probably have to struggle to appreciate the value of pluralism when a political majority legalizes same-sex marriages.

Although assessments of the inherent inertia of intolerance (cf. Gibson, 2011) differ, scholarly efforts have been devoted to understanding possible changes in this regard. According to learning theories, political tolerance may be fostered through participation in various institutional and social settings, such as schools, work places and civil society associations (Marquart-Pyatt & Paxton, 2007; Peffley & Rohrschneider, 2003; cf. Finkel, 2003). Although a touch of democratic romanticism may be present in optimistic expectations of this kind (cf. Adman, 2008), the basic mechanism is not necessarily enigmatic. If tolerance is not a congenital frame of mind, but rather a complex concept that has to be intellectually acquired (Sniderman, 1975; Sullivan, Marcus, Feldman, & Piereson, 1981), it should take some effort to abandon previously developed inconsiderate, perhaps even antagonistic, viewpoints. Exposing oneself to a diverse set of opinions—indirect, when acquiring information through educational material or when consuming (diverse) mass media, or direct, via personal experiences from interacting with other people—would assumingly help a person to appreciate democratic rights in a less egocentric fashion. If this is true, an interesting question is of course to what extent the more precise conditions of such learning may be disclosed. Specifically, what kind of mechanisms encourages more tolerant attitudes among individuals in a given social environment?

The idea that tolerance may be learned seems intuitively plausible. At the same time, however, significant inter-country differences remain to be explained (Peffley & Rohrschneider, 2003; Viegas, 2007, 2010; Weldon, 2006). Scholars have identified patterns (although results from different studies hardly correspond fully) linked to macro characteristics, such as socioeconomic development, political culture, and democratic transition. Put bluntly, citizens of more affluent countries, with a longer democratic history, tend to show higher acceptance for political rights of ‘disliked groups’ than citizens in less wealthy and recently democratized countries.^1^

Considering such findings, we obtain an analytical backdrop for the essential query of this study. Specifically, what is to be expected when people migrate from countries with differing (aggregate) levels of tolerance? From a neutral, so to speak, learning perspective, it seems reasonable that migration—due to the change of environment that migration by definition generates—could engender an increase as well as a decrease in political tolerance. The end result in this regard should reasonably depend on whether a migrant moves to a more or less tolerant setting. To our knowledge, however, systematic analyses of migration-related changes in political tolerance are rare in previous research.^2^

In an effort to contribute to this field of research, we set out to explore the case of immigrants in Sweden in this respect. Given that Sweden regularly rank very high in comparative studies on tolerance (Viegas, 2007, Weldon, 2006; cf. Kirschner, Freitag, & Rapp, 2011; Hadler, 2012), immigrants in this country may, in general, be expected to 1) have lower levels of tolerance, in comparison with the native population, but 2) become more tolerant over time, due to positive influences from contacts and observations in the new home country.

In the remainder of this paper, we first specify the theoretical model, according to which political tolerance may be fostered both through social interaction and via pure perceptually based assessment. In the following section, we describe the empirical data as well as our considerations and specifications of the central measures utilized in the study. Next, results from our empirical analyses are presented and evaluated in terms of the theoretical model. Finally, in the concluding section, we summarize our findings and discuss their implications for the prospects of political tolerance in contemporary multicultural welfare states.

## Learning to be tolerant—what should we expect?

To specify expectations derived from a perspective of learning on political tolerance, we may picture a politically intolerant person, being convinced that people with ‘unacceptable opinions’ should face tighter restrictions regarding democratic rights—at least when it comes to the freedom of publicly trying to convince opponents.^3^ Under what circumstances, then, would s/he reconsider such a stance?

Following more pessimistic outlooks in the literature, the question may seem to presume too much. Reviewing relevant studies on efforts to ‘foster’ desirable attitudes such as tolerance (for instance through government sponsored training programs; e.g. Finkel, 2003), Gibson does not seem to have much faith: ‘It may very well be that basic orientations toward foreign and threatening ideas are shaped at an early age, and, although environmental conditions can ameliorate or exacerbate such propensities, core attitudes and values are fairly resistant to change’ (Gibson, 2011, p. 420).

Moreover, based on evidence from a number of his own and other studies—performed in different regions of the world—Gibson (2011) concludes that tolerance is more pliable than intolerance. Providing respondents with arguments for tolerance, initially intolerant persons seemed to be highly reluctant to change their views; at least in comparison with the contrary scenario, in which tolerant respondents were much easier persuaded to reconsider their position. Hence, a fairly disheartening, though empirically supported, conclusion is that tolerance (similar to what conventional wisdom would say about general trust) is much more difficult to cultivate than to devastate.

Nevertheless, previous research also provides some support for the idea that individuals may change to become more politically tolerant. As mentioned, the essential suggestion of the learning model is that tolerance is a cognitively complex concept, the principles of which have to be grasped through some sort of internalised experiences (Marquart-Pyatt & Paxton, 2007; Peffley & Rohrschneider, 2003; cf. Gibson, 2011; Finkel, 2003). Tolerance, according to this assumption, should thus not be regarded as a congenital property. Rather, it may be developed among individuals in auspicious settings, where one eventually begins to embrace equal distribution of political rights. The settings in question have been labelled ‘socializing institutions’ (Marquart-Pyatt & Paxton, 2007, p. 93). Stressing the assumption that some kind of active cognitive effort is required, however, we will refer to these settings as ‘learning institutions’.

Compulsory as well as non-compulsory schooling of citizens probably provide the prime example of a learning institution when it comes to encouraging political tolerance. Education within a democratic society is assumed to provide knowledge and insights into different ideologies and viewpoints (Niemi & Junn, 2005; cf. Kokkonen, Esaiasson, & Gilljam, 2010).^4^ Hence, as a student, one should reach at least a rudimentary understanding of arguments behind the variety of political stances in society, including those that one does not approve of.

Aside from a curriculum-based broader understanding, however, tolerance is presumably learned also as a by-product of social interaction in schools. To the extent that the diversity of society (for example, in terms of ethnicity and religion) is mirrored in the composition of participants in educational institutions, students are provided with opportunities to interact with fellow students from different backgrounds. Following the optimism of the classic contact hypothesis in social psychology (Allport, 1954; cf. Pettigrew & Tropp, 2006), such pluralism may be expected to reduce prejudice and help people to see the benefits of tolerance. Hence, members of ‘out-groups’ may gradually be regarded with less suspicion, even if one does not adopt their opinions.

Although non-hostile contact is obviously a premise for the ‘learning’ we seek to conceptualise, our aim is also to more systematically identify demarcated social settings in which tolerance-building interaction potentially takes place. In the light if this, we picture ‘learning institutions’ as something broader than society’s formal educational system. Activities in other institutional settings may also provide opportunities to interact with people with different standpoints. Notably, learning may be expected to continue within the realms of working-life. Unlike educational institutions, which are supposed to convey democratic norms as a part of the curriculum, most employees are probably not subject to explicit democracy courses during their workdays. Still, similar to schools, workplaces provide contacts with other people. Also in absence of explicit learning, the job constitutes a social environment in which people interact and perhaps may obtain a deeper understanding of different perspectives (Mutz & Mondak, 2006; Pateman, 1970; but see Adman, 2008, for a critical appraisal).^5^ If work, hence, provides a potential seedbed for political tolerance, then lack of work, whether due to unemployment or retirement, all other things being equal, should reduce the likelihood of developing tolerant attitudes.

Considering additional potentially relevant institutional settings, organizational life may represent another platform for learning tolerance. The role of voluntary associations in civil society has a prominent place in democratic theory, at least since Mill (1861/1991); (cf. Strömblad & Bengtsson, 2017) developed ideas on organizations as schools in civic competence. By the same token, they habitually draw scholarly attention as potential sources of interpersonal trust, and hence social capital that may be reproduced outside of the associations as such (Putnam, 1993, 2000; cf. Paxton, 2007). Thus, although involvement in voluntary associations less frequently has been explicitly analysed as a predictor of political tolerance (but see Mutz & Mondak, 2006), civil society may very well represent an accompanying learning institution in this regard.^6^

Finally, scholars have pointed out that political tolerance reasonably could be encouraged through direct experience of utilizing democratic rights (Marquart-Pyatt & Paxton, 2007; Peffley & Rohrschneider, 2003; cf. Gibson & Duch, 1991). People who themselves are active in political life (for instance, by taking part in political meetings or by joining a political party) are assumed to become more prone to, in both word and deed, advocate political freedom also for one’s political opponents. Indeed, results from a study by Peffley and Rohrschneider (2003, pp. 252–254), encompassing seventeen countries, suggest that both democratic stability on the country level (taking differences in prosperity into account) and democratic activism on the individual level promote political tolerance among ordinary citizens. Similarly, Marquart-Pyatt and Paxton (2007, pp. 100–105) demonstrate that individual level democratic activism has such an expected positive effect, in the USA as well as in both Eastern and Western Europe. Admittedly, political involvement refers to activities, rather than to a specific institutional setting. Focusing on the potential importance of interacting with other people within this setting, however, we find it reasonable to include also political involvement under the heading of learning institutions for the purpose of this study.

Summing up the expectations, the learning model should be able to provide explanations for the initially presumed time-related increase in political tolerance among immigrants in Sweden. If migrants in general are influenced by the attitudes of the majority population in this respect, Sweden constitutes a promising most-likely case of an overall auspicious setting—given the country’s previously mentioned track record, based on tolerance surveys. Still, the processes involved might be somewhat more complex. Considering learning again, one may distinguish analytically between an *active* and a *passive* component, which may function simultaneously or separately. With this conceptualization, all four learning institutions described above (educational institutions, workplaces, voluntary associations, and political involvement) are in one way or the other expected to be influential due to ‘active learning’. Developing tolerant attitudes as a consequence of interaction in these institutional settings would in each case require personal attendance.

However, in an effort to develop the theoretical precision of the learning model, we also utilize the specific choice of population category in this study to provide some clue also on ‘passive learning’. Schematically, for the latter type of learning no actual personal interaction is necessary. Instead, one may assume that the overall social environment (in a given country, that is) provides knowledge and insights by a pure perceptual mechanism. By observing for instance political discussions and (non-hostile) political battles in a democratic society—via mass media as well as informal channels—the merits of tolerant attitudes may be perceived, even for a person with poor access to learning institutions.

**Fig. 1:**
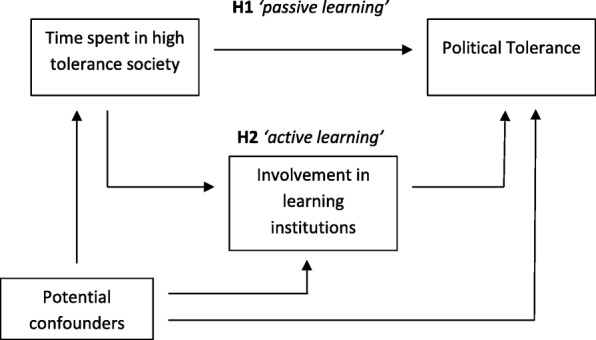
Analytical approach based on the learning model

Figure 1 provides a graphic representation of our analytical approach based on the learning model. Before operationalizing the variables (in the next section of the paper), the causal diagram highlights the principal relationships that ideally need to be investigated, in order to test the hypotheses.

As indicated by the graph, we put the learning model to test by formulation of two related hypotheses. The first hypothesis, H1, is based on the idea that Sweden may represent a high-tolerance society, in general thus providing an encouraging context in this respect for those who migrate to this country. Hence, H1 states that, among immigrants in Sweden, political tolerance increases with length of residence.

However, to buttress the developed version of the learning model also the second hypothesis, H2, should be empirically supported. Picturing a set of intervening variables in a hypothetical causal chain, H2 states that an initially positive effect of length of residence on political tolerance to a substantial extent is explained by greater involvement in learning institutions. As noted in Fig. 1, we consider such a path a case of active learning.

We find it reasonable to assume that passive and active learning at least to some extent take place simultaneously. Still, two ‘extreme’ outcomes are conceivable, corresponding to scenarios in which one but not both depicted paths turn out to be valid.

First, it may be that passive learning, implicating purely perceptually based information, is sufficient to influence individual level political tolerance. The empirical result compatible with such a possibility, we argue, would support the first hypothesis but not the second. In this case, an initially positive effect of length of residence remains observable irrespective of institutional involvement, and the involvement as such makes no difference in this regard.

Second, the opposite scenario is that a positive effect of length of residence is fully explained by relevant institutional involvement. In this case then, only the second hypothesis is supported, once the depicted paths are simultaneously evaluated. The reasonable conclusion would hence be that active learning is required, while passive learning in itself is insufficient.

Yet we find the portrayed causal model reasonable, the problem of determining causal direction is impossible to avoid completely with a survey-based rather than experimentally designed study. To some extent it may be the case that more politically tolerant immigrants, for whatever reason, tend to become more involved in host country learning institutions. As described in detail in the following section, we make use of extensive possibilities to control for confounding factors (cf. Fig. 1) in order to prevent a generous test of the hypotheses. We will return to the topic of inferential limitations in the concluding section of the paper.

## Measuring political tolerance and its causes—considerations and data

A common definition of political tolerance is ‘the willingness to respect political rights of individuals who belong to other groups’ (e.g. Finkel, Sigelman, & Humphries, 1999). Often it is added that this willingness should apply also to groups that one explicitly dislikes. Hence, political demonstrations and meetings conducted by one’s political opponents—and other groups one is against—should be accepted (Sullivan et al., 1982, p. 784).^7^

There are two dominating traditions as how to measure the concept in surveys. The first one is represented by Stouffer (1955), who in his seminal work focusing on the USA in the 1950s, examined individual’s tolerance for actions undertaken by certain ‘target groups’. More precisely, people were classified as intolerant if they denied civil liberties to socialists, atheists, or communists. This ‘fixed-group’ approach (Gibson, 2013) was later criticized for being confounded by the unpopularity of the selected groups. Sullivan and his colleagues (Sullivan, Piereson, & Marcus, 1979) therefore developed the content-controlled method, in which the respondents first are asked which group they like the least, and then whether they are willing to extend political rights to that group (such as arranging a political meeting). The groups are selected from all over the political spectrum. This approach has also been criticized in different ways; for example, for only considering left-wing and right-wing extremists, and for providing a too vague picture of the degree of a person’s tolerance level, as only one least-liked group is selected (e.g. Mondak & Sanders, 2003, pp. 495–496).

This criticism is taken into consideration when political tolerance is defined and measured here. A given respondent’s willingness to allow political rights to several specified groups in society will be investigated, and no attention is paid to whether the respondent would have expressed a dislike for a given group or not. Furthermore, tolerance will be regarded as a scale, ranging from full tolerance to full intolerance, depending on the number of groups one is tolerant or intolerant against (cf., the ‘breadth’ of intolerance; Gibson, 2006). Finally, we only include groups that undoubtedly should be politically respected, namely traditionally stigmatized (due to sexual orientation or illness) groups and ethnic minorities. The measure is then adequate, insofar as the political rights of these groups indisputably should be accepted, and may be considered as a ‘baseline tolerance measure’ from any reasonable democratic perspective.^8^

For the empirical analyses, we make use of the Swedish Citizen Survey 2003 (‘Medborgarundersökningen 2003’), which employed face-to-face interviews with a stratified random sample of inhabitants in Sweden (age 18–80).^9^ Admittedly, a more recent data set would have been preferable, all else being equal, not least in view of the extensive immigration to Sweden during the last decade. However, given the purpose of this study, we argue, the utilized survey represents the most complete source of information on political tolerance in Sweden, while also providing a rich set of measures of potential explanatory factors. Furthermore, the questionnaire of the survey included numerous questions on immigration-specific experiences and life circumstances. It should also be mentioned that, at the time the survey was administered, the immigrant population of Sweden already displayed a highly significant diversity—in terms of nationality and cultural background—as well as when it comes to reasons for migration.^10^

Our measure of *political tolerance* is based on four items. The respondents were asked whether they thought that homosexuals, people of a different race, people with AIDS, and drug addicts, respectively, should be allowed to hold public meetings. The answers were summarized in an additive index variable, measuring the number of groups to which one is tolerant.^11^ It was transformed to a scale 0–100, anchored in 0 = intolerance towards all groups and 100 = tolerance towards all groups (see Appendix for descriptive statistics of all variables used in the analyses).

The primary independent variable, *time in Sweden,* measures a respondent’s length of residence in the new home country. The measure is quite detailed as it takes into account the number of years as well as months the respondent has been living in Sweden (thus also taking into account temporary periods abroad). Expecting a diminishing rate of return of time in this regard—a learning effect should reasonably be more pronounced for relatively recent immigrants, than for those who already have spent several decades in the country—the variable was logarithmically transformed prior to the analyses.

Turning to variables capturing involvement in learning institutions, *post-migration education* measures the number of years spent in combined full-time schooling and occupational training in Sweden.^12^ As for the potential importance of working life, the dummy variables *weak labour force attachment* (coded 1 for respondents that are unemployed, or on disability pension, or not working for other reasons; and 0 otherwise) and *pensioner* (coded 1 for those who are retired; and 0 otherwise) separates respondents in the corresponding categories from those who are employed, and thus may take part in social interaction at workplaces.

Regarding possible acquirement of tolerance in civil society organizations, we include a measure of *associational activity*, based on questions on engagement in 28 different types of voluntary associations. The measure includes a wide-ranging array of recreational organizations, interest and identity organizations, as well as ideological organizations, and has been summarized in an additive index variable.^13^

Finally, we capture respondents’ practical use of democratic rights by the measure of *political participation,* thus incorporating conventional forms of participation as well as more recently recognized non-parliamentary ways to bring about societal change (cf. Barnes et al., 1979; Stolle, Hooghe, & Micheletti, 2005; Teorell, Torcal, & Montero, 2007). Again, we use an index variable consisting of items on a total of 19 different modes of participation included in the survey (such as voting, party activities, personal contacts, protests, and political consumerism).^14^ Analogous to the expected non-linear effects of length of residence, the variables associational activity and political participation were logarithmically transformed as well.

As earlier indicated, accounting for a series of possibly confounding factors is necessary. The demographic factors age and gender have sometimes been found to correlate with tolerance. Younger individuals usually show higher levels of tolerance than older, and some studies have found men to be more tolerant than women (e.g. Bobo & Licari, 1989; Golebiowska, 1999; cf. Togeby, 1994). The variable *female* is coded 1 for women and 0 for men, and *age* is the respondent’s age the year the interview took place. As for potentially important migrant-specific variables, we also control for possible acquirement of Swedish citizenship (the corresponding variable is coded 1 if the respondent is a Swedish citizen, and 0 otherwise).

Furthermore, potential differences due to the reason for migration, are captured by the variable *refugee* (coded 1 for people who migrated to Sweden either because they were refugees themselves, or because they accompanied or joined a relative with refugee status; and 0 for those who came to Sweden for other reasons, such as for work or studies). Finally, we constructed a set of dummy variables separating immigrants in three categories based on their respective origins in different regions of the world. The first category ‘west’ (used as a reference category the statistical analyses in the next section) consists of immigrants from western and Anglo-Saxon countries, specifically, other Scandinavian countries, North-western Europe, Australia, Canada, New Zeeland, and the USA. Next, the second category ‘east’ consists of immigrants from Eastern and Southern Europe. Finally, the third category ‘south’ consists of immigrants from Africa, the Middle East, Asia, and Latin America. Admittedly, this trichotomy is crude, but Myrberg (2007) has nevertheless demonstrated its empirical validity when it comes to conditions for immigrants in Sweden.^15^

## Time-related tolerance—empirical results

In this section, our version of the learning model is put to an empirical test. Before evaluating corresponding regression equations, a brief look at the data suffice to conclude that initial expectations are born out, when it comes to differences between immigrants in Sweden and native Swedes. Specifically, given the measure of political tolerance on a 0–100 scale, the mean tolerance level proved to be 87.8 in the first mentioned population category and 93.4 in the latter. The difference as such is statistically significant (*p* < 0.01), and yet approximately 6 percentage points does not seem to indicate a huge gap in absolute terms, we argue that it is nonetheless substantially interesting. In view of the rather ‘cautious measure’ of political tolerance—not demanding more than respect for a few, arguably vulnerable, groups’ right to hold political meetings—a high level of tolerance is reasonably anticipated, along with a low variability of scores (indeed, the standard deviation of the entire sample proved to be a fairly modest 17.5).

Moreover, a somewhat closer look on differences due to origin reveals that migrants from some regions of the world, in comparison with others, clearly tend to report lower levels of political tolerance in Sweden. Echoing previous findings in comparative research (e.g. Marquart-Pyatt & Paxton, 2007, p. 99), we find that non-European immigrants from countries in Africa, Asia and Latin America as well as immigrants from Eastern Europe (the ‘south’ and ‘east’ categories, respectively, specified in previous section) score almost 10 percentage points lower than native Swedes. While these differences also are highly statistically significant (*p* < .001, in both cases), there is no distinguishable difference in political tolerance between the Sweden-born population and immigrants from the Western world.

Considering a well-established association between country of origin and length of residence in Sweden (cf. SCB, 2013), there are obvious reasons to try to disentangle a possible time-related impact of a general ‘high-tolerance exposure’ in Sweden from attitudinal inertia related to the country of origin.

The empirical results summarized in Table 1 provide us with some guidance in this effort. As the table shows, two regression models were estimated, based on the same sample of respondents with a migrant background. Model 1, to begin with, includes the primary independent variable time in Sweden, along with the series of potential confounders which otherwise may have created a spurious association. Studying the result for the time variable, we note that the first hypothesis (H1) receives some empirical support. Controlling for demographic and other potentially influential differences among immigrants, the statistically significant and positive coefficient suggests that time spent in a high tolerance society indeed stimulate the development of politically tolerant attitudes. Since the OLS estimation is based on a logarithmic transformation of the length of residence, a possible interpretation of the coefficient is that a 10% increase in the time spent in Sweden would be associated with somewhat less than a 1-point increase on the political tolerance score. Considering the non-linear relationship, time may thus after all have a fairly substantial impact in this respect; that is, among people that rather recently have migrated to Sweden.

**Table 1:** Predicting political tolerance among immigrants in Sweden, considering time-related differences and involvement in learning institutions

	Model 1	Model 2
Time in Sweden (log)	7.1 (2.6)^***^	4.0 (2.7)
Female	−0.6 (1.8)	−0.9 (1.7)
Age	−0.6 (0.4)	−0.8 (0.4)^*^
Age squared	0.001 (0.004)	0.006 (0.005)
Pre-migration education	0.3 (0.2)	0.4 (0.3)
Swedish citizen	−0.4 (2.2)	−1.0 (2.4)
Refugee	−2.1(2.3)	−2.5 (2.2)
*Origin* (West = ref.)		
East	−7.3 (2.7)^***^	−5.5 (2.7)^**^
South	−10.3 (2.4)^***^	−8.8 (2.4)^***^
Post-migration education		0.6 (0.2)^**^
*Labour market position* (Employed = ref.)		
Weak labour force attachment		- 5.3 (3.1)^*^
Pensioner		−7.0 (5.5)
Associational activity (log)		0.04 (0.7)
Political participation (log)		4.4 (1.5)^***^
Constant	95.3	94.3
*N*	696	696
*R*^*2*^	0.086	0.130

Entries are ordinary least-squares (OLS) estimates with standard errors in parenthesis. The sample is weighted to be representative of people who have immigrated to Sweden. The dependent variable political tolerance runs from 0 (no group is politically tolerated) to 100 (all four groups are politically tolerated)

*Statistical significance:* ***p < .01, **p < .05, *p < .10

Evaluating the results regarding the control variables as well, we note that the previously mentioned differences between immigrants from different regions of the world essentially remain. Interestingly, however, taking origin into account, immigrants who have acquired Swedish citizenship do not report higher political tolerance than non-Swedish citizens. Similarly, we find no discernible tolerance differences between refugees and those who have immigrated for other reasons.^16^ Contrary to some previous studies, we also do not find any differences in political tolerance related to gender or age, among immigrants in Sweden. Furthermore, although the length of pre-migration education seems to be positively associated with respect for political rights, the coefficient is statistically insignificant. In contrast, as we will examine shortly, this is not the case when participation in educational institutions in Sweden is scrutinised.

Moving forward, the results based on estimation of Model 2 provide a basis for evaluating the second hypothesis (H2). Recall that H2 (once H1 has received empirical support) stated that a positive time-related effect on political tolerance to a substantial degree should be explained by expected positive links via involvement in learning institutions (cf. Fig. 1 above). However, before further examining these links empirically we may—in line with expectations—observe a sizable decrease of the regression coefficient of time in Sweden (when Model 2 and Model 1 results are compared). Moreover, the coefficient is no longer statistically significant. This result thus supports H2: A considerable part of the difference in political tolerance between immigrants with varying length of residence in Sweden seems to be explained by variations in institutional involvement. True, the fairly low R-squared values for both models suggest that a series of other factors may influence political tolerance. However, although further research is needed to uncover details when it comes to determinants of tolerance (including possible differences in terms of the relative strength of influencing factors between immigrants and the native population in Western democracies), this does not prevent us from evaluating the fruitfulness of the suggested causal model.

Focusing, hence, on substantial interpretation in the light of the model, we may infer that a relatively short period in Sweden does not provide enough opportunities for interaction within the four learning institutions. This, in turn, also appears to have detrimental consequences for political trust. Among immigrants in Sweden, other things being equal, taking part in post-migration education, having a job rather than being unemployed, and participating politically, represent activities that in each case seem to encourage political tolerance.^17^ With the exception of activity in voluntary associations (a variable for which no direct effect is discernible in Table 1),^18^ the results thus suggest that institutional involvement provides active learning in line with theoretical assumptions—either by means of education in a more narrow sense, or as by-product of social interaction with people of different backgrounds.^19^

On the other hand, though, the final estimation does not provide any evidence supporting the idea that passive learning simultaneously takes place. Considering that the positive effect on political tolerance of length of residence in Sweden is, in effect, explained by the better possibilities to participate in learning institutions, simply ‘observing’ a more tolerant society does not seem to be sufficient.

Yet stronger relationships might be found, should more comprehensive and fine-grained measures of institutional involvement become available, it is worth noting that the small set of such factors accounted for in this analysis is sufficient to analytically remove the *direct* path between length of residence in Sweden and political tolerance. Hence, although passive learning is a theoretically conceivable contextual effect in the explored setting, the empirical data suggest that such an effect is too weak to be identified.

**Fig. 2:**
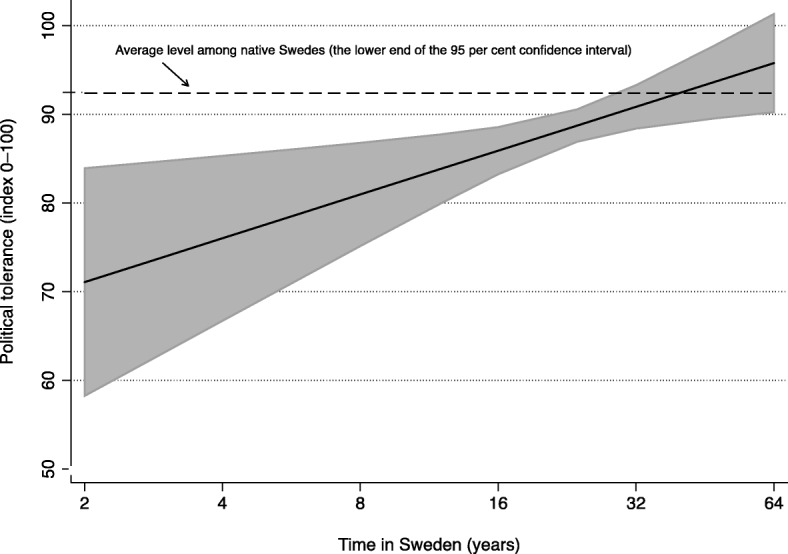
Predicted levels of political tolerance among immigrants. The graph is based on the estimated relationship of political tolerance and length of residence in Sweden, according to Model 1 in Table 1 (i.e., controlling for background characteristics, but before taking involvement in learning institutions into account). The grey shaded area displays 95 percent confidence intervals for the predictions

The overall positive time-effect is graphically illustrated in Fig. 2, displaying variation in predicted levels of political tolerance among immigrants with different lengths of residence in Sweden. Importantly, the positive relationship depicted by the solid line in the graph does not take the learning institution effects into account (i.e., the predictions are based on the Model 1 estimation results above). Thus, the total importance of time in Sweden is illustrated—including the indirect positive path, via institutional involvement. Considering the log-transformation of the time-variable (manifested in the scale of the x-axis, roughly representing the actual empirical range of the variable), we notice that, in general, immigrants eventually tend to adapt to the average level of political tolerance among native Swedes (represented by the dashed line in the graph).^20^ However, yet time gradually encourages active learning, the adaptation is still expected to need a considerable number of years. According to our prediction equation, political tolerance levels are on par for native Swedes and immigrants that have been residing in Sweden for about 30 years (judging from the position on the x-axis corresponding to the point of intersection in the graph, where the dashed line crosses the confidence interval of the solid line).

Generally speaking, then, immigrants in Sweden are prone to develop a greater respect of democratic rights over time. However, such a development is slow, and seems to require active input via educational institutions and workplaces, and preferably also that immigrants make use of their democratic rights by active political involvement.

## Conclusion and discussion

In this paper, we have explored the idea that more politically tolerant attitudes may be developed as a consequence of exposure to a high-tolerant social environment. Taking the theoretical point of departure in a learning model, we have tried to study the role of time in this respect. Specifically, time has been evaluated as an indicator of overall exposure to (previously empirically established) high level of tolerance in Sweden, and in terms of anticipated positive relationships between time and participation in important social settings in which tolerance may be fostered and encouraged.

To assemble an empirical test bed, we used survey data including rich and detailed information on a representative sample of immigrants in Sweden. Utilizing a dynamic feature of these data, we empirically evaluated the importance of length of residence in Sweden—and thus residence in a comparatively speaking high-tolerance society. In harmony with the hypotheses put forward, we found that political tolerance among immigrants in Sweden in general seems to increase over time in the new home country. Controlling for an extensive series of possible confounding factors, a more extensive time-period in Sweden seems to encourage a more comprehensive recognition of political rights among people who have migrated to this particular country. Moreover, in concert with theoretical expectations, our analyses suggest that the positive time-related effect is substantially mediated through participation in ‘learning institutions’ within the realms of education, working-life, and political involvement. Hence, a possible prediction would be that an initially intolerant person, who migrates to Sweden, is likely to adopt more broad-minded and permissive attitudes regarding political rights over time. Such a positive scenario, from a democratic point of view, would then result from increasingly better possibilities for this immigrant to meet and appreciate tolerant opinions, as a by-product of educational, work-related and political activities.

Although we find this learning effect to be both intuitively reasonable and theoretically well anchored, the limitations of the cross-section data should of course be acknowledged. In the study we may only infer that time has had the described impact in the light of ‘present’ levels of institutional involvement and political tolerance. In absence of panel data, such a causal inference should naturally be made with caution. Similarly, a possible distortion due to differing response rates between sub-categories of immigrants may potentially lead to bias, should we have failed to control for exogenous variables correlated (in hitherto unknown ways) with survey participation as well as with participation in social settings and political tolerance. In this paper, we have utilized Swedish data, to analyse how an overall politically tolerant context may influence attitudes among people with prior experiences from, in general, less tolerant contexts. In order to probe the validity of the suggested model, along with the generality of our findings, we certainly encourage further studies; preferably based on a more extensive set of immigration host countries as well as time-periods.

This may also provide further important knowledge from a policy implication point of view. The results from our study may induce some optimism insofar as political tolerance—indispensable in a democratic society—may be developed also among adult newcomers in a democracy. Still, the results suggest that considerable time is demanded for this to take place. If policies could be designed in a way that maximise possibilities of integration—while also reducing segregation that prevents intercultural social interaction—more fast-track featured routes of learning tolerance might be established.

Yet our findings, as such, may be regarded as a questioning of previous assumptions of rigidity when it comes to levels of (in-)tolerance acquired early in life, one should bear in mind that our evidence is based on previous, rather than present, conditions of integration in Swedish society. Clearly, the possibilities to get access to learning institutions—and, presumably important, to experience equal treatment once inside such institutions—may very well be different in time-periods mapped in future studies. Thus it should not be taken for granted, neither in Sweden nor elsewhere, that immigrants currently meet integrative institutions on a regular basis, or that time for tolerance will be provided in the future.

## Appendix

**Table 2:** Descriptive statistics

	Minimum	Maximum	Mean	Standard deviation
Political tolerance (index)	0	100	88.2	21.1
Time in Sweden (years)	2.2	73.5	27.5	15.2
Female	0	1	0.55	0.50
Age	20	82	49.1	15.2
Pre-migration education (years)	0	25	9.1	5.4
Post-migration education (years)	0	24	4.1	5.0
Weak labour force attachment	0	1	0.14	0.35
Pensioner	0	1	0.16	0.37
Swedish citizen	0	1	0.73	0.44
Associational activity (index)	0	9	1.04	1.28
Political participation (index)	0	19	3.29	3.09

Weighted *N* = 696

**Table 3:** Indexes for associational activity and political participation – relative frequency distributions (per cent)

No. of reported activities	Associational activity	Political participation
0	40.9 (40.9)	11.4 (11.4)
1	35.4 (76.4)	22.5 (34.0)
2	11.9 (88.3)	16.3 (50.3)
3	6.2 (94.5)	13.6 (63.9)
4	2.7 (97.2)	10.6 (74.4)
5	1.6 (98.9)	10.3 (84.7)
6	0.9 (99.8)	3.5 (88.2)
7	0.0 (99.8)	2.6 (90.9)
8	0.2 (100)	1.7 (92.5)
9		1.0 (93.5)
10		2.1 (95.6)
11		1.2 (96.9)
12		1.2 (98.1)
13		0.7 (98.8)
14		0.1 (98.9)
15		0.4 (99.3)
16		0.4 (99.8)
17		0.0 (99.8)
18		0.1 (100)

Weighted *N* = 696. Entries are relative frequencies with cumulative percentages within parentheses

**Table 4:** Political tolerance – principal component analysis

Items (groups that should/should not be allowed to hold public meetings)	Loadings (component 1)
Homosexuals	0.75
People of a different race	0.68
People with AIDS	0.77
Drug addicts	0.65

Entries reproduce the component matrix from a principal component analysis. Applying the Kaiser criterion, only one component was extracted (with Eigenvalue = 2.03), explaining 51% of the variance in the variables included

**Table 5:** Predicting political tolerance: alternative index

	Model 1	Model 2
Time in Sweden (log)	3.5 (2.3)	1.2 (2.3)
Post-migration education		0.2 (0.3)
*Labour market position* (Employed = ref.)		
Weak labour force attachment		−4.3 (2.5)*
Pensioner		−2.5 (4.5)
Associational activity (log)		−0.4 (0.6)
Political participation (log)		6.0 (1.3)***
Constant	90.8	86.8
*N*	696	696
*R*^*2*^	0.035	0.067

Entries are ordinary least-squares (OLS) estimates with standard errors in parenthesis. The sample is weighted to be representative of people who have immigrated to Sweden. The alternative political tolerance index (cf. Table 4 in Appendix) utilised in this analysis includes items referring to three more groups: ‘left-wing extremists’, ‘right-wing extremists’ and ‘racists’. All control variables (cf. Table 1 above) are also included in the respective regression equations, but not reported here. The dependent variable political tolerance runs from 0 (no group is politically tolerated) to 100 (all seven groups are politically tolerated)

Statistical significance: ****p* < .01, **p* < .10

**Table 6:** Predicting political tolerance: separate dimensions of political participation

	Model 1	Model 2
Time in Sweden (log)	7.1 (2.6)***	4.2 (2.2)*
Political participation: voting		3.3 (1.8)*
Political participation: party activities		2.5 (5.2)
Political participation: contacting		−0.1 (4.2)
Political participation: reactive manifestations		3.1 (2.8)
Political participation: proactive manifestations		5.6 (4.5)
Constant	95.3	94.9
*N*	696	696
*R* ^*2*^	0.086	0.105

Entries are ordinary least-squares (OLS) estimates with standard errors in parenthesis. The sample is weighted to be representative of people who have immigrated to Sweden. In this analysis, the overall political participation index (cf. note 14) is replaced with variables representing five dimensions of political participation listed in table. Reactive manifestations refers to the following items: ‘has signed a petition’; ‘has boycotted certain products’; ‘has deliberately bought certain products for political, ethical or environmental reasons’; and ‘has donated money’. Proactive manifestations refers to the following items: ‘has worn or displayed a campaign badge or sticker’; ‘has participated in a public demonstration’; and ‘has raised funds’. All control variables are also included in the respective regression equations, as well as the variables representing the other learning institutions (cf. Table 1 above) but not reported here. The dependent variable political tolerance runs from 0 (no group is politically tolerated) to 100 (all four groups are politically tolerated)

Statistical significance: ****p* < .01, * *p* < .10

**Table 7:** Predicting involvement in four learning institutions

Dependent variable	Effect of time in Sweden	*R* ^*2*^	*N*
1. Post-migration education	2.204 (0.317)***	0.63	696
2. Weak labour force attachment (vs. working)	−0.064 (0.035)*	0.05	696
3. Associational activity	0.544 (0.142)***	0.05	696
4. Political participation	0.295 (0.068)***	0.08	696

Entries are ordinary least-squares (OLS) estimates with standard errors in parenthesis. The sample is weighted to be representative of people who have immigrated to Sweden. Here, the four learning institutions were set as dependent variables in separate regression analyses, to examine the respective effect of time in Sweden (cf. Fig. 1) on each of them. All control variables are also included in the respective regression equations (cf. Table 1 above) but not reported here. The coding of the learning institution variables is described in the methodological section of the paper. For reasons of simplicity, the variable ‘pensioner’ is excluded when estimating the time effect on working-life participation (thus an OLS linear probability model is used in analysis no. 2, as the dependent variable in this case is a dummy separating unemployed and those on disability pension from those who are in paid work)

Statistical significance: ****p* < .01, * *p* < .10

## Footnotes

1 Furthermore, the study by Peffley and Rohrschneider (2003), taking socioeconomic development and democratic longevity into account, also suggests that tolerance is encouraged in federalist (rather than centralist) political systems.

2 The migration perspective notwithstanding, compared with a series of other socio-political attitudes and behavioural patterns (e.g., social/institutional trust and political participation) political tolerance does not seem to be ‘mapped’ with the same frequency in large-scale comparative surveys; hence, relevant data are not as readily available for scholars.

3 It may very well be less challenging for the politically intolerant person to agree that all citizens, regardless of their opinion on more or less controversial matters, should have the right to vote in general elections. The ‘silent’ act of casting a vote is not associated with visible persuasion in the same vein as, for example, a public demonstration. Thus, the latter mentioned act will more often be considered as provoking, or outright dangerous, in the eyes of an opponent.

4 Still, it is worth mentioning that schools, unsurprisingly, differ in how well they manage to transmit democratic norms to their students (Jormfeldt, 2011).

5 Analogous to the question of diversity in schools, the actual level of pluralism of, e.g., political opinions at a given workplace, reasonably depends on the heterogeneity of the staff (Mutz & Mondak, 2006).

6 The study by Marquart-Pyatt and Paxton (2007) acknowledges the potential importance of civil society organizations. Due to data restrictions, however, their analyses include a (dichotomous) measure of membership, but no measure of actual activity.

7 A closely related concept is social tolerance, i.e. whether other (disliked) groups are considered as socially equal (e.g. accepted as neighbours). The relationship between various manifestations of political and social tolerance is clearly interesting, yet beyond the scope of this study.

8 Although we consider the tolerance index chosen to be theoretically as well as methodologically defensible, we have experimented with an index in which also less indisputable groups are included; specifically, ‘left-wing extremists’, ‘right-wing extremists’ and ‘racists’. As shown in the Appendix, using this index in regression analyses discloses similar results to those reported in Table 1, although fewer variables (including ‘time in Sweden’) turn out to be statistically significant.

9 Principal investigators were Karin Borevi, Per Strömblad, and Anders Westholm at the Department of Government, Uppsala University. The fieldwork was carried out in 2002 and 2003 by professional interviewers from Statistics Sweden. The interviews averaged about 75 min in length, and translations of the questionnaire were available in six large minority languages. Funding was supplied by the Bank of Sweden Tercentenary Foundation, and by the Government Commission of Inquiry on the Political Integration of Immigrants in Sweden. The overall response rate was 56.2%. All analyses in this paper have been conducted with proper adjustments for the stratified sampling procedure employed—through which immigrants from developing countries (often having a refugee background) were over-represented in the sample, while immigrants from Nordic and Western European countries were under-represented. Importantly, since we find it very hard to suggest a reasonable weighting procedure in order to try to adjust for potential bias due to varying response rates between different sub-categories of immigrants, the adjustment mentioned strictly refers to a sample-weight based correction of unequal selection probabilities. In total, 858 respondents in the full sample (originally selected on the basis of official registry data) had at some point in time migrated to Sweden.

10 According to an integration policy report from the Swedish government in 2002 (thus corresponding well to the time of the survey fieldwork) it was estimated that roughly one-third of the foreign born residing in Sweden had immigrated as refugees, one-third for family reasons and one-third as labour-market migrants; the same proportional structure was also found for region of origin, with equal shares of immigrants coming from Nordic countries, from other countries in Europe, and from countries outside of Europe (Sveriges Riksdag, 2002).

11 Results from a principal component analysis suggest that it is reasonable to consider the items as one-dimensional. A single factor is retained based on the Kaiser criterion, explaining 51% of the variance, and with factor loadings varying 0.65–0.77 (see Appendix).

12 As a control variable, also pre-migration education, accomplished outside of Sweden is taken into account.

13 Specifically, the different types of associations are: ‘Sports club or outdoor activities club’; ‘Youth association (e.g. scouts, youth clubs)’; ‘Environmental organization’; ‘Association for animal rights/protection’; ‘Peace organization’; ‘Humanitarian aid or human rights organization’; ‘Immigrant organization’; ‘Pensioners’ or retired persons’ organization’; ‘Trade union’; ‘Farmer’s organization’; ‘Business or employers’ organization’; ‘Professional organization’; ‘Consumer association’; ‘Parents’ association’; ‘Cultural, musical, dancing or theatre society’; ‘Residents’ housing or neighbourhood association’; Religious or church organization’; ‘Women’s organization’; ‘Charity or social-welfare organizations’; ‘Association for medical patients, specific illnesses or addictions’; ‘Association for disabled’; ‘Lodge or service clubs’; Investment club’; ‘Association for car-owners’; ‘Association for war victims, veterans, or ex-servicemen’; and ‘Other hobby club/society’.

14 Political participation has long been regarded as a multidimensional concept (e.g., Verba & Nie, 1972), but for reasons of simplicity an index variable consisting of items on all different participation forms are used here. A scree-test, based on a principal component analysis, in fact provides some support for treating political participation as a one-dimensional phenomenon (for a similar approach, see e.g. Verba, Schlozman, & Brady, 1995, especially p. 544). The items included in the index are, besides voting in the local elections (2002), whether one—in trying to bring about improvements or to counteract deterioration in society—during the last 12 months: has contacted a politician; has contacted an association or an organization; has contacted a civil servant on the national, local or county level; is a member of a political party; has worked in a political party; has worked in a (political) action group; has worked in another organization or association; has worn or displayed a campaign badge or sticker; has signed a petition; has participated in a public demonstration; has participated in a strike; has boycotted certain products; has deliberately bought certain products for political, ethical or environmental reasons; has donated money; has raised funds; has contacted or appeared in mass media; has contacted a lawyer or judicial body; has participated in illegal protest activities; or, has participated in political meetings. However, separate analyses have also been undertaken where the different participation items are sorted according to a standard multidimensional approach (separating between voting, party activities, contacting, and different forms of manifestations). The results thus obtained are very similar to those presented in this paper (see Appendix). Analysing voting, it should be noted that also non-citizen (adult) immigrants in Sweden are eligible to vote in local elections, provided that certain conditions regarding length of residence are fulfilled (different conditions apply for EU and non-EU citizens).

15 Moreover, as a robustness check in additional analyses, we replaced the trichotomy with 21 different world-regions of origin (thus separating migrants from, e.g. east and west Africa, south and southeast Asia, the Middle East and so on). The results (available from the authors upon request) do not differ substantially from those reported in this paper.

16 We excluded, as yet another robustness check, observations for respondents reporting that they had migrated in order to study (who turned out to be very few). This did not have any substantial impact on the results.

17 It deserves to be mentioned that the negative effect of the variable ‘weak labour force attachment’ to some extent might indicate also that unemployed immigrants fail to develop more tolerant attitudes if such a social position tend to trigger suspicions of discriminatory treatment and thus resentment. Still, as shown in the table the tendency is similar also among pensioners (although the corresponding coefficient is not statistically significant); that is, among those that only because of age no longer may enjoy working-life based learning.

18 As for the non-discernible effect of associational involvement, this may to some extent reflect less potent influences among ethnic associations, in terms of promoting political networks, as previously suggested by Strömblad and Adman (2010).

19 Further analyses revealed statistically significant and positive effects of time in Sweden on participation in all four learning institutions (see Appendix). We also re-ran the models in Table 1 using ordered logit analysis. The findings (available from the authors upon request) do not differ substantially from those reported in Table 1.

20 As noted in Fig. 2, native Swedes’ average level of political tolerance is conservatively estimated, since the dashed line corresponds to the lower end value of a 95% confidence interval (specifically, the value is 92.4, whereas the previously mentioned mean estimate in this population category is 93.4).
